# Distribution of *ACE *insertion/deletion (I/D) polymorphism in Iranian populations

**Published:** 2015-06

**Authors:** Mostafa Saadat

**Affiliations:** Department of Biology, College of Sciences, Shiraz University, Shiraz, Iran

**Keywords:** *ACE*, Iran, Polymorphism, Population genetics

## Abstract

Angiotensin converting enzyme (ACE; OMIM: 106180) has an important role in the conversion of angiotensin I to angiotensin II and degradation of bradykinin. Genetic polymorphism I/D (rs4646994) in the gene encoding *ACE *has been well defined. To get more insight into the genetic structure of Iranian populations, the distribution of the *ACE *I/D polymorphism among Iranians was compared with each other and with other populations. Prevalence of the D allele was 0.5886 (95% CI: 0.5725-0.6047) in Iran. There was significant difference between Iranian populations (*x*^2^=27.7, df=6, P<0.001). The major part of this difference was due to difference between Zahedan study and the other populations, as by removing this population, the heterogeneity between populations, remarkably decreased (*x*^2^=10.15, df=5, P=0.071). The D allele showed high frequency in Iran which is similar to Caucasians.

## INTRODUCTION

Angiotensin converting enzyme (EC 3.4.15.1; ACE; OMIM: 106180), is a circulating and membrane bound enzyme. The ACE has an important role in the conversion of angiotensin I to angiotensin II and degradation of bradykinin, a potent vasodilator, which mediates a wide range of cellular functions in different tissues. There is a genetic polymorphism (rs4646994) which is characterized by an insertion/deletion (I/D) within intron 16 in the gene. The D allele has been associated with a higher mRNA level and higher ACE activity [1, 2]. Studies have been demonstrated that this polymorphism is associated with several multifactorial diseases [3, 4].

Iranian population is one of the most heterogeneous populations of the world [5, 6]. To get more insight into the genetic structure of Iranian populations, the present study was carried out.

## MATERIALS AND METHODS

Eligible studies having raw data on the I/D polymorphism of *ACE *in healthy Iranian populations were identified by searching the database in the PubMed (National Center for Biotechnology, National Library of Medicine), DOAJ (Directory of Open Access Journals), ISC (Islamic world Science Citation Center), and SID (Scientific Information Database) for relevant reports published before December 2014 using the following search terms: "*ACE*", "polymorphism", “ins/del”, “I/D”, and "Iran". Furthermore, references cited in the retrieved articles were screened to trace additional relevant studies. We found 11 studies eligible for our analysis [7-17]. A Chi-square test was performed to determine whether the study samples demonstrated Hardy-Weinberg equilibrium for the mentioned polymorphism. Comparison between populations for prevalence of the D allele was done using a Chi-square test. A probability of P<0.05 was considered statistically significant.

## RESULTS AND DISCUSSION

The study samples of the published articles for prevalence of the genotypes of the I/D polymorphism were at Hardy-Weinberg equilibrium. Table 1 shows the prevalence of the D allele in seven Iranian populations using published articles [7-17]. In overall prevalence of the D allele was estimated 0.5886 (95% CI: 0.5725-0.6047) in Iran. There were significant difference between Iranian populations (*x*^2^=27.7, df=6, P<0.001). A majority of this difference corresponds to difference between Zahedan study with the other populations, because removing this population, the heterogeneity between populations, remarkably decreased (*x*^2^=10.15, df=5, P=0.071).

Based on the published articles, there were significant differences in terms of the D allele frequency between the three major ethnic groups. The frequency of the D allele varied among populations, suggesting an ethnic distribution. It seems that the prevalence of the D allele increased from east to west. The prevalence of the D allele was lower among Asian populations (about 25-40%) [3, 4] compared with the Caucasians (generally about 40-60%) [3, 4] and Africans (more than 60%) [18, 19]. The allelic prevalence of the D allele in our sample (about 62.6%) seems to be more similar to the Caucasians than the Asians.

Previous reports on other genetic polymorphisms showed that Iranian gene pool revealed intermediate frequency in comparison with European Caucasians and Asians [6]. However, the present study did not support that conclusion. Some evolutionary forces may be involved for this difference. Arabian populations such as Kuwait, UAE, and Oman showed high prevalence of the D allele (61.0-75.0%) [18, 20]. We know that there was migration between Arab communities and Iranian populations. High prevalence of the D allele among Arabs and their migration into Iranian populations, might be considered at least in part, as the reason for not having the intermediate frequency of the D allele in Iranian population.

**Table 1:** Distribution of the D allele of *ACE* I/D polymorphism among several populations

**Province**	**Ethnicity**	**Number of subjects**	**D (%)**	**References**
Ardabil	Azaris	97	63.6	7
Fars	Persians	363	62.8	8
Kermanshah	Kurds	100	54.3	9
Sistan va Balochestan	Mixed	132	46.6	10
Tehran	Mixed	744	59.0	11-14
West Azarbaijan	Kurds	207	54.4	15
West Azarbaijan	Azaris	230	59.6	16, 17

## Notes

**Acknowledgements: **This study was supported by Shiraz University.
